# The Interplay between Hydrogen Sulfide and Phytohormone Signaling Pathways under Challenging Environments

**DOI:** 10.3390/ijms23084272

**Published:** 2022-04-12

**Authors:** Muhammad Saad Shoaib Khan, Faisal Islam, Yajin Ye, Matthew Ashline, Daowen Wang, Biying Zhao, Zheng Qing Fu, Jian Chen

**Affiliations:** 1 International Genome Center, Jiangsu University, Zhenjiang 212013, China; yusufzai.pathan786@hotmail.com (M.S.S.K.); faysal224@yahoo.com (F.I.); zhaoby@ujs.edu.cn (B.Z.); 2 Key Laboratory of Forest Genetics and Biotechnology, Ministry of Education of China, Co-Innovation Center for the Sustainable Forestry in Southern China, Nanjing Forestry University, Nanjing 210037, China; yajinye@njfu.edu.cn; 3 Department of Biological Sciences, University of South Carolina, Columbia, SC 29208, USA; mashline@email.sc.edu; 4 State Key Laboratory of Wheat and Maize Crop Science, College of Agronomy, Henan Agricultural University, Zhengzhou 450002, China; dwwang@henau.edu.cn

**Keywords:** hydrogen sulfide, biotic stress, abiotic stress, salicylic acid, abscisic acid, jasmonic acid, ethylene, auxin, phytohormones

## Abstract

Hydrogen sulfide (H_2_S) serves as an important gaseous signaling molecule that is involved in intra- and intercellular signal transduction in plant–environment interactions. In plants, H_2_S is formed in sulfate/cysteine reduction pathways. The activation of endogenous H_2_S and its exogenous application has been found to be highly effective in ameliorating a wide variety of stress conditions in plants. The H_2_S interferes with the cellular redox regulatory network and prevents the degradation of proteins from oxidative stress via post-translational modifications (PTMs). H_2_S-mediated persulfidation allows the rapid response of proteins in signaling networks to environmental stimuli. In addition, regulatory crosstalk of H_2_S with other gaseous signals and plant growth regulators enable the activation of multiple signaling cascades that drive cellular adaptation. In this review, we summarize and discuss the current understanding of the molecular mechanisms of H_2_S-induced cellular adjustments and the interactions between H_2_S and various signaling pathways in plants, emphasizing the recent progress in our understanding of the effects of H_2_S on the PTMs of proteins. We also discuss future directions that would advance our understanding of H_2_S interactions to ultimately mitigate the impacts of environmental stresses in the plants.

## 1. Introduction 

The in-depth understanding of mechanisms/processes involved in plant growth and development is critical for improving crop quality and productivity, as well as the development of more stable and climate-resilient crops. Due to their sessile nature, plants have evolved several adaptive mechanisms for survival. Among them, phytohormones are complex signaling factors that regulate a myriad of physio-biochemical processes to maintain optimum growth, development, and performance [1]. The synthesis and level of hormones could vary significantly in different plant tissues, during different developmental stages, and under different environmental conditions [2]. Furthermore, there is less knowledge about the coordination of the spatial and temporal distribution of plant hormones and how these dynamic processes trigger diverse responses in plants [3].

**Figure 1:**
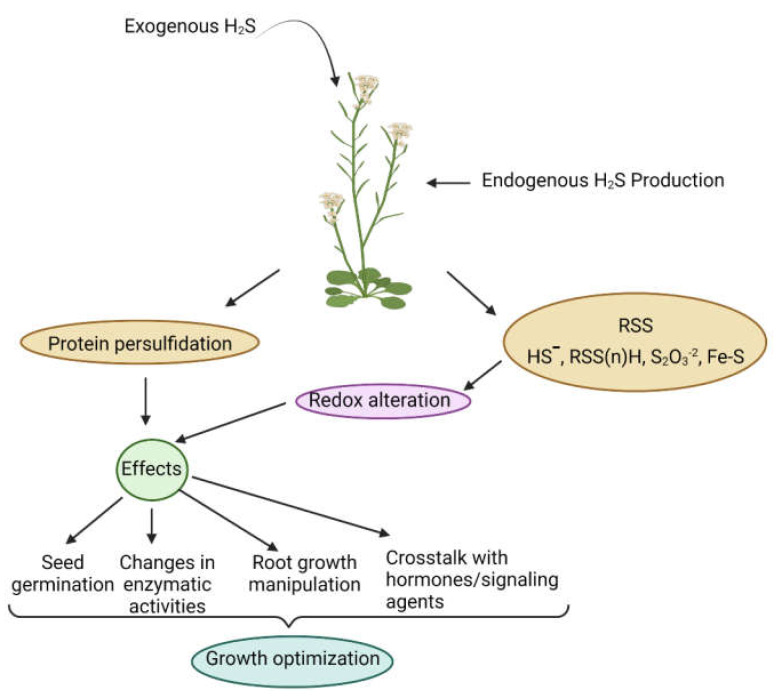
Overview of hydrogen sulfide (H_2_S) production and the regulation of several physiological, metabolic, and morphological processes by H_2_S to optimize growth in plants.

Recently, numerous investigations have revealed hydrogen sulfide (H_2_S) as one of the critical components in various acclimation processes in plants under normal and stressful conditions (Figure 1). H_2_S is a colorless, lipophilic, toxic, volatile, inflammable, and water-soluble gas with a pungent odor, similar to that of rotten eggs. Amidst the emergence of life on Earth approximately 3.8 billion years ago, H_2_S acted as a major energy source; however, H_2_S-dependent organisms disappeared after a burst of oxygen [4]. Nevertheless, the biogeochemical sulfur cycle was preserved in organisms and is presently limited to some vital metabolic and signaling events [5,6]. H_2_S receives extensive attention in the animal field due to its multiple physiological and pathophysiological functions in different organs due to clear and well-established experimental models/approaches [7]. However, it was not until recently that the roles of H_2_S in plants have gained the attention of scientists due to the involvement of H_2_S in adverse stress conditions via regulation of gene expression, post-translational modifications (PTMs), and crosstalk with other gaseous signals and phytohormones [8,9].

The fine-tuned interaction of H_2_S with other gaseous signaling biomolecules and hormones orchestrates molecular, metabolic, and physiological adaptive responses and permits the plants to respond properly to changing environmental conditions. In this review article, we will explain the central role of H_2_S in the regulation of various physiological and molecular processes. We will also discuss how hormonal homeostasis plays a crucial role in stress conditions and how H_2_S synergistically/antagonistically regulates the biosynthesis and degradation of the associated plant hormones and modulates their signaling to generate adaptive responses in plants.

## 2. H_2_S Biosynthesis in Different Organelles and Associated Enzymes

**Figure 2:**
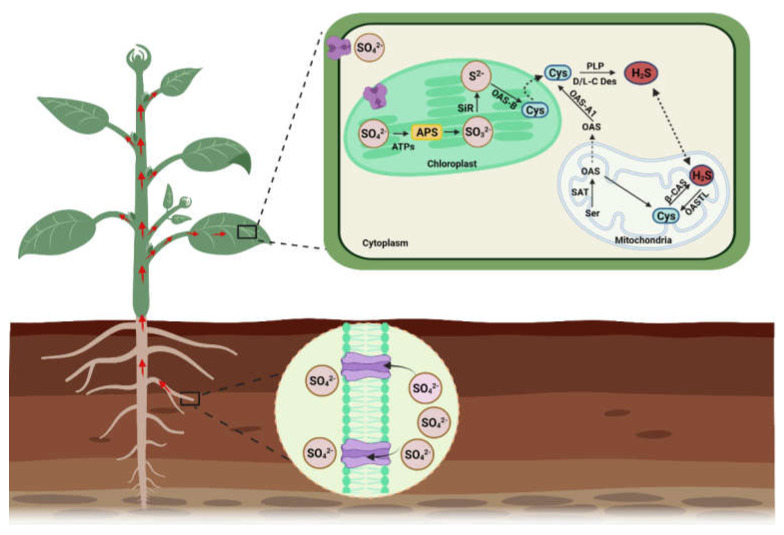
H_2_S biosynthesis in plants. In plants, sulfate (SO_4_^2−^) is transported from the roots, which is then distributed to all parts of the plant through the xylem vessels. SO_4_^2−^ entering the cells is assimilated in the chloroplasts and mitochondria. In chloroplast, SO_4_^2−^ is reduced to sulfite (SO_3_^2−^) by APS reductase after it is activated to APS. Under the catalysis of SiR, the sulfite is then reduced to sulfide (S^2−^) using six electrons transferred from ferredoxin. As a result, sulfide is produced, which is used to produce cysteine. The OASTL enzyme catalyzes the synthesis of cysteine along with O-acetylserine. The enzyme CDes and pyridoxal 5-phosphate (PLP) participate in degrading cysteine to generate H_2_S. In mitochondria, serine acetyltransferase (SAT) catalyzes the conversion of serine (Ser) into OAS and produces cysteine, which is converted to H_2_S via the catalytic activity of β-cyanoalanine synthase (β-CAS).

Plant roots absorb sulfate (SO_4_^2−^), which is reduced into H_2_S via the action of APS reductase (adenosine-5′-phosphoryl sulfate reductase) and SiR (sulfite reductase). H_2_S is later transformed into cysteine amino acid via catalysis of O-Acetylserine (thiol) lyase (OASTL), as a final step of sulfate assimilation in plants (Figure 2). In *A. thaliana*, cytosolic OAS-A1 (At4g14880), the plastid OAS-B (At2g43750), and the mitochondrial OAS-C (At3g59760) are considered true OASTL because they incorporate an O-acetylserine (OAS) and sulfide into cysteine synthesis [10,11,12]. The presence of functional OASTL was also identified in pollen [13]. Additionally, plant cells contain nutritional sulfur (SO_4_^2−^) and SO_2_ (collected from the atmosphere) that is consequently converted into SO_3_^2−^ and is used to produce H_2_S in the presence of ferredoxin and APS reductase [14,15]. In salt-stressed tobacco plants, malfunction of SiR leads to decreased H_2_S production, correlating with less availability of SO_2_ on account of stomatal closure. This series represents the functional role of SiR in H_2_S metabolism under stress conditions [16].

H_2_S is also synthesized in the chloroplasts and mitochondria when cysteine is reduced by cysteine desulfhydrase (CDes) and β-cyanoalanine synthase (β-CAS), respectively (Figure 2). Genetic and molecular evidence indicated that mitochondrial isoforms of CAS are CYS-C1 (At3g61440) and OAS-C (At3g59760), and chloroplastic isoforms of CAS are OAS-B (At2g43750) and SCS (At3g03630) [17]. The cytosolic release of H_2_S is dependent upon the functioning of D/L cysteine desulfhydrases (L/D-CDes). Several L-CDes of the Arabidopsis plant are well characterized and are involved in the breakdown of L-cysteine to sulfide, NH_3,_ and pyruvate [18,19,20]. However, D-CDes are completely different proteins and belong to the pyridoxal 5-phosphate (PLP)-dependent enzyme superfamily, and its activity is PLP dependent [21,22]. The model plant Arabidopsis contains two putative *D-cysteine desulfhydrases* (*D-CDes*) genes (At1g48420 and At3g26115) [21,22,23], while two *D-CDes* are also functionally characterized in rice (*OsDCD1* and *OsLCD2*) and some other crops [24,25]. The D-cysteine desulfhydrases 2 carry out the decomposition of both L- and D-Cystine into H_2_S. Accumulating evidence signifies that NifS-like L-CDes are also involved in the generation of H_2_S. The presence of H_2_S in plant peroxisomes and its interaction with catalase is also observed; however, the synthesis mechanisms and involved enzymes are still unknown [26].

The mitochondria play a vital role in the catabolism of H_2_S and maintain its steady-state levels in cells. In mitochondria, H_2_S is generated during cyanide detoxification through the catalysis of β-CAS. The functional mitochondria isoform of CAS is CYS-C1 (At3g61440), which catalyzes the conversion of cysteine and cyanide into hydrogen sulfide and β-CAS and maintains optimum levels of cyanide to prevent phytotoxicity [27]. This yielded H_2_S is converted back into cysteine via mitochondrial OASTL (OAS-C, At3g59760), which will again be used in the detoxification of cyanide. This process is considered a cyclic pathway of cysteine generation via H_2_S consumption in mitochondria [28]. Under stress conditions, excess accumulation of H_2_S raises the pH of mitochondria, leading to the conversion of H_2_S into hydrosulfide ions (HS^−^). Excess accumulation of H_2_S also prevents the loss of H_2_S from mitochondrial membranes and maintains H_2_S homeostasis (Figure 2). The environmental cues also modulate the endogenous H_2_S biosynthesis by stimulating desulfhydrase activities in plant cells [18].

In plastids, the reduction of sulfate to sulfide and its incorporation into the OAS is executed as an entry point of reduced sulfur to plant metabolism for growth and development via a photosynthetic sulfate assimilation pathway [18,29]. The OAS interaction with serine acetyltransferase (SAT) forms a cysteine synthase complex (CSC), which generates demand-driven synthesis of cystine in plant cells [30,31]. Subsequently, the breakdown of cysteine in the chloroplast generates H_2_S due to the catalysis of DES1 and L/D-cysteine desulfhydrase (Figure 2). The generation of H_2_S in chloroplasts acts as a signaling molecule because it substantially impacts cellular metabolism by limiting the rate of photosynthesis.

The peroxisome is an essential single membrane-bound organelle involved in the metabolism of reactive nitrogen species (RNS), including H_2_S [26,32,33]. Recent studies demonstrated the presence of H_2_S in plant peroxisomes [34]. Some studies speculated that peroxisomes have the capacity to transform sulfite to sulfate under the catalysis of Sulfite oxidase (At3g01910) in *A. thaliana*. Presently, no enzymatic source for H_2_S metabolism has been observed in the peroxisome of Arabidopsis, and tomato [34,35,36]; the mechanism of H_2_S production in peroxisome is still obscure. The H_2_S characterization study in *Solanum lycopersicum* showed the localization of OASTL9 in the peroxisome, which exhibited upregulation under different developmental stages and pathogenic bacterial treatments [36].

In the plant, several additional enzymes are also involved in H_2_S synthesis, and most of the H_2_S in the cell is produced during the necessary consumption of cysteine. For example, At5g28030 encodes a cysteine synthase (CS)-like protein that degrades L-cysteine and produces H_2_S [28]. This protein is also localized in the cytoplasm as AtDES1 (desulfhydrase). The homolog of this protein in *Brassica napus* (BnDES) is also involved in the breakdown of cysteine [37]. However, AtDES1 homolog in rice (OsLCD2) exhibits cysteine biosynthesis activity [38]. The Arabidopsis nitrogen fixation-like 1 and 2 (At5g65720; At1g08490) also use L-cysteine as a substrate and produce H_2_S during the synthesis of L-alanine in the cytosol [20,28,39]. This diversity in enzymatic functioning and discrepancies in their substrates’ catalyzation may allow the plants to calibrate endogenous H_2_S levels according to their requirements and external prompts.

## 3. Role of H_2_S in the Modulation of Abiotic Stress Responses

**Figure 3:**
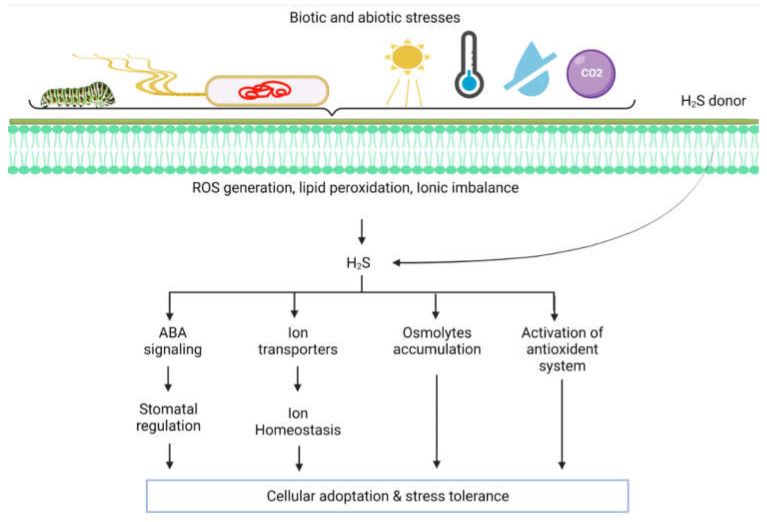
Multiple environmental stressors can induce endogenous hydrogen sulfide (H_2_S) production in plants. The H_2_S production mediates the different physiological processes in plants by undergoing interaction with plant hormones and other cellular entities to maintain homeostasis under normal and stressful conditions.

H_2_S plays a vital role in protecting plants against several abiotic stressors. Environmental stress factors such as salinity, drought, waterlogging, high temperature, excessive light, heavy metals, and chilling could adversely affect plant growth and development (Figure 3) [40,41,42,43]. Generally, under most stress conditions, plants reduce uptake of CO_2_ due to the closure of stomata and limiting CO_2_ fixation. This condition causes alternation in cell metabolism due to restricted photosynthetic capacity that leads to the generation of reactive oxygen/nitrogen species (ROS/RNS) [44,45,46,47,48,49,50]. H_2_S directly regulates the cysteine (Cys) residues’ persulfidation via posttranslational modification (PTM), allowing the H_2_S to regulate protein functioning through persulfidation [51,52]. For example, APX protein was persulfidated in different compartments of cells (cytosol, chloroplasts, mitochondria, and peroxisomes) in Arabidopsis [26,53,54,55]. These findings indicate that the ROS-induced toxicity in stressed plants is regulated by H_2_S-mediated persulfidation post-translationally via triggering the ROS scavenging enzyme activities [56].

### 3.1. Application of H_2_S in Plant Drought Responses

During osmotic stress, improved water status of plants is a vital survival strategy that is achieved via accumulating osmolytes to maintain normal hydration levels. Exposure to drought stress or PEG-induced osmotic stress in plants enhances the accumulation of osmolytes such as proline and glycine betaine to maintain normal water status in stressed plants. However, sometimes the accumulation of these osmolytes fails to maintain adequate water status due to the severity of osmotic stress [50,52]. The endogenous stimulation of H_2_S regulates the proline synthesizing enzyme via stimulating the expression of 1-pyrroline-5-carboxylate synthetase, and by inhibiting the activity of the proline-degrading enzyme. On the other hand, H_2_S also triggers the activity of glycine betaine biosynthesis enzymes (aldehyde dehydrogenase), which reduce the osmotic stress and assist the plants in enhancing osmotic pressures to improve water uptake and relative water content in vital tissues [57,58]. The pre-exposure of SO_2_ to drought-stressed wheat plants showed a pronounced increase in endogenous H_2_S. This inflation may be caused by the conversion of SO_2_ into the SO_3_^2−^ and decomposition of L-/D-Cys, which generates enough H_2_S to initiate drought adaptive responses in the stressed seedling. However, when hypotaurine (HT; H_2_S scavenger) was applied on SO_2_-pretreated seedlings, reduced content of H_2_S and severe symptoms of drought toxicity appeared in seedlings. In addition, endogenous generation of H_2_S via pretreatment of SO_2_/NaHS, fully activated the antioxidant enzymes (SOD, CAT, and POD) and reduced the production of H_2_O_2_ and MDA content in drought-stressed plants [41,47,59,60]. The endogenous H_2_S modulation in plants also activated the expression of transcription factors (TFs) such as *ERF1*, *NAC69*, and *MYB30* [41,61]. The findings of several studies indicated that TF NAC69 could confer resistance in drought-stressed plants via the H_2_S mediated ABA signaling pathway. Additionally, the upregulation of TFs such as *ERF1* and *MYB30* may activate signal transduction pathways and regulate stress-responsive gene expression profiling under drought stress conditions [62,63,64]. Since the application of H_2_S scavengers inhibited the transcript abundance of *ERF1*, *NAC69*, and *MYB30* in wheat plants under drought stress conditions, there must be direct involvement of H_2_S in the regulation of stress-related TFs in response to drought stress [61,65,66]. Some studies also recognized that H_2_S signaling in response to drought stress influences the functioning of ABA biosynthesis genes such as *NCED2*, *NCED3*, and *NCED5* and suppresses the ABA catabolic genes (*ABA8ox1*, *ABA8ox2*, and *ABA8ox3*), which is consistent with ABA accumulation in drought-stressed plants [47,50].

### 3.2. Role of H_2_S in the Alleviation of Metal Stress

Under metal toxicity, plants modulate several metal/metalloid ions from toxic to less toxic forms, such as reduction of arsenate (AsV) to arsenite (ASIII), and *hexavalent chromium* (*Cr*^(VI)^) to less toxic trivalent *Cr*^(*III*)^, and sequester these metal ions via thiols (GSH) and phytochelatins (PCs) ligands [64]. These metabolites (GSH and PCs) actively participate in the intracellular redox balance and metal tolerance capacity of crop plants and prevent the cells from entering programmed cell death or necrosis phases [67,68]. Due to metal-induced oxidative stress, the intracellular redox becomes oxidized, decreasing levels of reduced molecules such as NADH/NADPH and allowing apoptosis or necrosis to be initiated. The endogenous production of H_2_S or exogenous application of H_2_S donors assists in maintaining the levels of GSH and phytochelatins in the plant to sustain optimum redox balance and the sequestration of toxic metal ions into the vacuoles [41,69]. The GSH and PCs are sulfur enriched compounds, whereas, in sulfur metabolism, metabolites such as sulfite, H_2_S, cysteine, and GSH are highly interconnected, and depletion of GSH during metal toxicity could potentially accelerate cysteine breakdown and ultimately enhance the GSH and H_2_S supply to the cell [16,67]. In several published studies, it is observed that the mitigation effects of H_2_S under different abiotic stresses and metal excess conditions are related to the upregulation or superior maintenance of redox-active compounds such as ASA-, GSH, and PCs [40,67,69,70]. This finding of these studies provides compelling evidence that modulation of endogenous H_2_S during stressful conditions could help the plant to maintain or reduce the loss of intracellular glutathione, which supports the overall redox positive state of the cell and verifies that H_2_S has an important influence on cell functions under stressful conditions [41,70,71].

H_2_S not only overcomes ROS-induced toxicity in metal exposed plants but also plays an effective role in the inhibition of metal transport and absorption. H_2_S has the ability to alter chemical forms of metal ions into insoluble phosphate compounds, which decreases metal toxicity and movements [72]. However, the metal reduction capacity of H_2_S is much lower than GSH, cysteine, phytochelatins, and metallothioneins [73]. H_2_S mediated reduction in metal transport/immobilization is usually associated with downregulation of metal transporters or secretion of chelating compounds to prevent the further translocation of metal ions to the sensitive tissues or uptake from the root zone. For example, in several crop plants, exogenous application of H_2_S intensifies the citrate secretion and expression of citrate transporters, so the non-toxic complexes of citrate with Al^3+^ could be formed in the rhizosphere [74,75,76]. Similarly, H_2_S also suppresses pectin methyl esterase activity, which suppresses Al^3+^ binding sites by reducing negative charge in root cells, which has direct implications for Al^3+^ tolerance [77,78]. In the case of Cd metal, H_2_S triggers the expression of phytochelatin synthase (*PCS*) and the Cd-ATPase gene to effectively chelate and transport metal ions into the vacuoles through the help of HMT transmembrane transporter channels [79]. The L-DC-mediated H_2_S accumulation modulates root pectin content with a lower degree of methylation to facilitate the binding of Cd^2+^ to the cell wall, which ultimately diminishes its further translocation from root to shoot and toxicity symptoms in exposed plants [80]. In Arabidopsis, exogenous application of H_2_S activated the generation of Cr^6+^ binding peptides, such as phytochelatins and metallothioneins, to carry toxic Cr^6+^ to insensitive regions mediated by compartmentalization [81,82]. Based on these studies, we infer that H_2_S plays a pivotal role in the chelation of heavy metals for inactivation and later sequesters them into the vacuole to increase the metal stress tolerance of plants.

### 3.3. Effect of H_2_S on Plant Salt Tolerance

Salinity is a major constraint limiting agriculture productivity due to poor irrigation practices and continuous climate fluctuations [83]. Saline stress imposes both osmotic stress and ionic toxicity, which retard plant growth and productivity. The unregulated accumulation of sodium (Na^+^) hinders water and nutrient uptake and induces water deficit conditions for plants. Furthermore, an excessive amount of Na^+^ and chloride (Cl^−^) accumulation in plants disturbs ionic homeostasis. The depolarization of membranes leads to the loss of potential stress mitigating ions such as K^+^ and Ca^2+^ and induces changes in transpiration rate, photosynthesis, oxidative stress, etc. [84,85,86]. Saline stress in plants reinforces several physiological, molecular, and metabolic disorders that completely inhibit plant growth [87,88,89]. The maintenance of ionic homeostasis and a lower cytosolic Na^+^/K^+^ ratio is critical for salt adaptation and tolerance. It is observed that several Na^+^/K^+^ ion transporters and stress-responsive gene activation pathways are interconnected with plant hormones because stress and growth hormones are spatially involved in mediating salt-stress signaling and maintaining the balance between stress responses and growth in plants [83,87,88]. In this regard, H_2_S biosynthesis and signaling are implicated in saline stress tolerance in plants. [90,91,92,93]. Several studies demonstrated that exogenous application of H_2_S reduces the uptake of Na^+^ and increases the accumulation of K^+^ that untimely preserves an optimal Na^+^/K^+^ ratio for the plant’s vital functioning. [90,91,92,93]. It is proven via pharmacological studies that when H_2_S scavengers were applied to the salt-stressed plants, the depletion of endogenous H_2_S aggravated the saline stress symptoms and increased the Na^+^/K^+^ ratio and cytosolic concentration of Na^+^ in studied plants. These studies also highlighted that H_2_S application significantly maintains K^+^ homeostasis in plants by preventing K^+^ leakage by reducing oxidative stress-mediated lipid peroxidation and membrane depolarization. [90,91,92,93]. At the molecular level, it was observed that H_2_S regulated the activity of SKOR (outward rectifying K^+^ channel) by inhibiting its expression and preventing the loss of K^+^ into the xylem under saline stress conditions. However, when H_2_S scavengers (DL-propargylglycine or HT) were applied to the plants, SKOR expression was not compromised. [90,91,92,93]. Similarly, the K^+^ retention during saline stress conditions normalizes H^+^-ATPase, because H^+^ gradient-mediated H^+^-ATPase activity repolarizes the PM to accelerate potassium influx and sodium efflux [90,91,92,93]. This repolarization occurs because H_2_S is involved in the stimulation of gene expression and phosphorylation-mediated upregulation of H^+^-ATPase activity under salinity [94,95]. This observation suggests that H_2_S shows the implication of K^+^ uptake and its homeostasis via upregulating the K^+^/Na^+^ antiport system through modulating H^+^-ATPase activity [42,91,92,95]. Besides this, AKT1 (inward rectifying potassium channels) is located in root epidermal tissue [96], and HAK5 (potassium transporter) gene is located in the tonoplast and the PM [96]. These genes are also coupled with maintaining K^+^ and plant resistance to salt. The exogenous application of H_2_S donors improved the transcript expression of *AKT1* and *HAK5* and total K content in the salt-challenged *Brassica napus* plant [97]. Similarly, NaHS induced H_2_S promoted the expression of *HvAKT1* and *HvHAK5* in roots of barley seedlings under salinity [8]. All these findings advocate that the potential increase in H_2_S and its signaling is a positive regulator of K^+^ homeostasis and maintenance of the Na^+^/K^+^ ratio during saline stress in plants [8,95,98,99].

The (SOS) pathway is critical for the exclusion of Na^+^ under saline stress conditions. SOS1 is involved in the long-distance transport of Na^+^ from roots to shoots [95,100]. The increase in transcript abundance of *SOS1* favors the accumulation of SOS1 proteins in the PM, which triggers the exclusion of Na^+^ from cells and minizines the Na^+^ load in the cytosol [60]. The H_2_S application under alkaline and normal salt stress conditions stabilizes the mRNA level of *SOS1,* which leads to the reduced Na^+^ content in the roots of cultivated apple plants [101]. SOS1 is regulated by the H^+^ gradient provided by PM H^+^-ATPase. Several studies identified that H_2_S positively influences the gene expression and phosphorylation of PM H^+^-ATPase under salinity [102]. In pharmacological experiments where endogenous H_2_S production was inhibited, the expression level of *SOS1* and related Na^+^ antiporters were downregulated, and salinity tolerance of plants was compromised due to unregulated accumulation of Na^+^ in sensitive tissues [100]. The PM H^+^-ATPase on the membranes of vacuoles also regulates the expression and activation of the Na^+^/H^+^ antiporter, because the compartmentalization of Na^+^ ions into the vacuoles is an alternative solution to decrease the Na^+^ induced toxicity in cells [42,103]. The H_2_S application greatly induces the transcript accumulation of *NHX2* and *VHA-β* genes (Na^+^/H^+^ antiporter) in salt-exposed plants. This finding also advocates that Na^+^ caging in vacuoles is influenced by H_2_S signaling [8,85]. Meanwhile, for the regulation of Na^+^/K^+^ homeostasis, H_2_S also controls the H_2_O_2_ mediated activity of PM-bound NADPH oxidases [104]. For instance, PM NADPH oxidase inhibitor (diphenyleneiodonium chloride) suppressed the H_2_S mediated increase in H_2_O_2_ in the root of Arabidopsis under salinity. The application of ROS scavenger (N,N’-Dimethylthiourea) abolished the H_2_S mediated H_2_O_2_ production in salt stress plants due to the Na^+^ uptake being high in salt-stressed plants from the absence of H_2_S mediated activation of NADPH oxidase [104]. This conclusion indicates that H_2_O_2_ might act as a downstream signal for H_2_S-mediated Na^+^/K^+^ homeostasis [85,104,105]. The findings of these studies demonstrate that H_2_S regulated signaling influences the activity of H^+^-ATPase and the expression of PM Na^+^/H^+^ antiporter that enhances the salt tolerance by maintaining Na^+^/K^+^ homeostasis in plants [85,106].

## 4. Crosstalk of H_2_S with Signaling/Phytohormones under Changing Environmental Conditions

Phytohormones, or plant growth regulators (PGRs), are the most significant signaling molecules, synthesized in specific locations within plants, and can be translocated to different parts to regulate stress responses [106]. PGR such as abscisic acid (ABA), auxins (IAA), brassinosteroids (BRs), cytokinins (CK), gibberellins (GA), jasmonic acid (JA), and salicylic acid (SA) help the plants to overcome numerous biotic/abiotic adversities by triggering physiological and molecular responses [107,108]. H_2_S, which acts as an endogenous gasotransmitter, is recognized in relevance with other signaling molecules such as NO [109], ROS [110], H_2_O_2_ [111], CO [112], and plant hormones such as ABA [113], JA [114], GA [115] and ethylene.

H_2_S in plants exhibits a dual role, either disseminated as pernicious cellular repercussion or as credible signaling molecules depending upon stress conditions. A study discovered that H_2_S operates downstream of NO and helps decrease oxidative stress during salt stress in tomatoes. H_2_S helps minimize postharvest ripening and senescence in bananas because it inhibits ethylene signaling as well as mitigating oxidative stress [115]. Additional studies revealed that H_2_S regulates NADPH oxidase (RBOH) activity, leading to ROS accumulation [116]. Simultaneously, the concentration of phosphatidic acid generated via phospholipase D [117,118] is also modulated by H_2_S, which helps further to inhibit the cellular signaling pathway [1]. In Arabidopsis, H_2_S operates upstream of the MAPKs pathway, and both of these work parallelly under cold stress conditions [119]. Various developmental processes such as organogenesis, seed germination, and the advent of senescence are spurred by H_2_S produced from sodium hydrosulfide (NaHS) and morpholin-4-ium 4-methoxyphenyl (morpholino) phosphinodithiolate (GYY4137) [119,120]. As a signaling molecule, H_2_S participates in several cross-talk networks amid H_2_O_2_, NO, CO, and phytohormone ABA during different stress conditions [121]. It is evident that signaling molecules such as H_2_S interplay an essential role in several stages of plant development because of the interaction between H_2_S and numerous phytohormones. In the future, genes involved in governing the new signaling molecules such as H_2_S could be targeted to develop a genetically improved crop.

### 4.1. Crosstalk of H_2_S and Abscisic Acid (ABA)

Plants modify ABA levels continually in response to changing physiological and environmental conditions, while bioactive ABA levels are sustained through a fine balance between generation and catabolism [45,86,87]. Several ABA receptors are involved in signal perception and transduction [45]. Earlier studies revealed that the interaction of H_2_S with ABA receptor genes implied that H_2_S regulates ABA signaling via influencing ABA receptors [45,122,123]. H_2_S application in drought-stressed plants upregulated the expression of potential ABA receptors such as *RCAR* (*The regulatory component of ABA*), *ABAR* (*abscisic acid receptor*), *PYR1* (*pyrabactin resistant protein*), *GTG1* (*GPCR-type G proteins*), and *CHLH* (*H subunit of the Mg-chelatase*) [45,124]. Some studies point out that ABA regulates many physiological processes, and H_2_S sometimes regulates these responses in a similar way [45,113,124]. Exogenous application of ABA triggers the endogenous production of H_2_S, suggesting complex crosstalk between two signaling molecules exists under drought stress conditions [45]. Similarly, under heat stress, ABA could trigger the accumulation of endogenous H_2_S and act as a new downstream gaseous signaling molecule that regulates ABA-induced stress responses in heat-stressed plants [45].

In plants, stomatal closure or opening is regulated by guard cells. The plant hormone ABA regulates the function of several ion channels in an ABA-dependent manner to control stomatal closure and opening [124,125,126,127,128]. A wealth of literature provides ample evidence that H_2_S regulates stomatal aperture in various plant species, and it may have implications for ABA-dependent stomatal closures in plants under stressful conditions [124]. The earlier study of Wang et al. [129] illuminated this underlying mechanism and revealed that exogenous application of H_2_S activates the S-type anion currents in guard cells of Arabidopsis. Concurrently, the elevated level of free Ca^2+^ is a prerequisite for its activation [129]. H_2_S triggers Ca^2+^ waves in guard cells. In guard cells, Ca^2+^ sensing is perceived by a heterotrimeric G-protein β-subunit (AGB1) that collaborates in Ca^2+^ induced stomatal closure in Arabidopsis [130]. Ca^2+^ ions also activate SLAC1 by stimulating CPK (calcium-dependent protein kinase) activity. It was observed that lower concentrations of ABA partially impaired stomatal closure in *CPK* quadruple mutant plants; however, higher concentrations of ABA effectively close stomata. The application of Ca^2+^ chelator (1,2-bis(*o*-aminophenoxy) ethane-*N*,*N*,*N*,*N*-tetraacetic acid (BAPTA) completely inhibited the ABA-mediated activation of anion channel in guard cells and prevented the ABA-induced stomatal closure [131,132]. These studies showed that H_2_S and ABA are signaling components in stomatal closure in plants.

**Figure 4:**
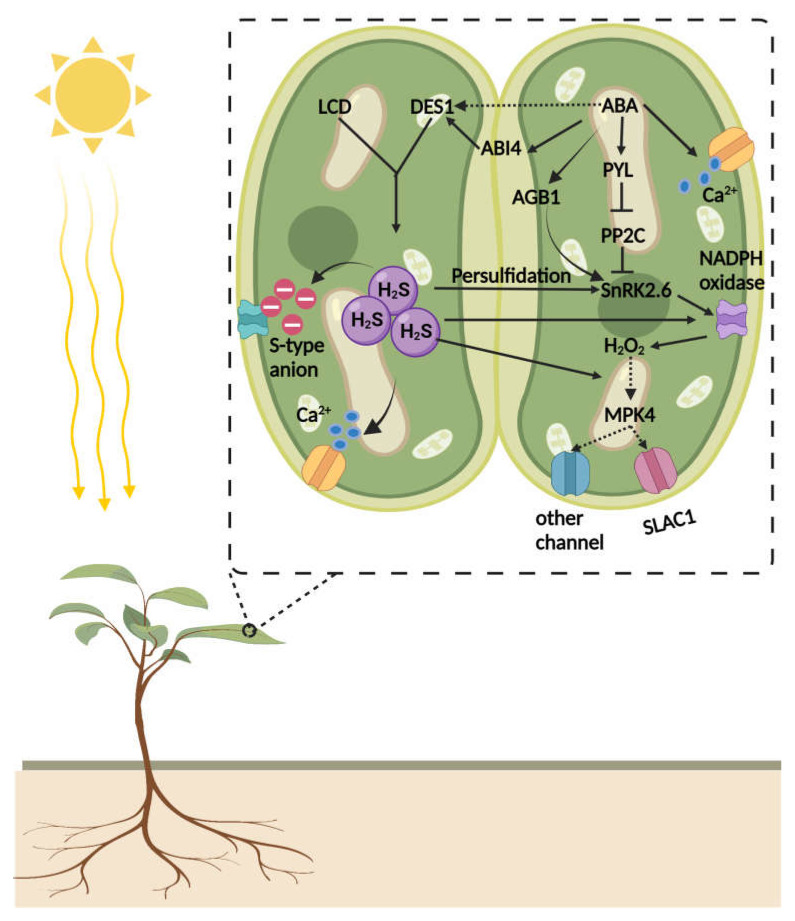
Under normal conditions, ABA receptors (PYR/PYL/RCAR) bind to the PP2Cs and inhibit the activity of SnRK2.6, which deactivates NADPH oxidase, SALC1, and other ion channels to reinforce the normal functioning of stomata. Under water-stressed conditions, ABA signaling stimulates ABA receptors (PYR/PYL/RCAR) that lead to the activation of SnRK2.6, which triggers SLAC1 and NADPH oxidase to produce H_2_O_2_ and regulate stomatal movements. During drought stress, ABA signaling increases the biosynthesis of H_2_S via persulfidation of ABI4-mediated activation of DES1 transcription. The burst of H_2_S in guard cells activates the S-type anion and spikes the Ca^2+^ wave alongside strong persulfidation of SnRK2.6. The persulfidated SnRK2.6 robustly phosphorylates SALAC1 and NADPH oxidase to produce a long-lasting burst of ROS to modulate water efflux in guard cells to close stomata, similarly to the way that ABA induces stomatal closure.

A recent study demonstrated that H_2_S mediated persulfidation of SnRK2.6/OST1 in response to ABA signaling initiated stomatal closure (Figure 4). In guard cells, SnRK2.6/OST1 acts as a core component of ABA signaling that controls stomatal movements, and its function is tightly regulated by H_2_S-mediated PTMs. Under certain physiological conditions, ABA induces the generation of H_2_S by activating DES1 in the guard cell. The accumulation of H_2_S persulfidates SnRK2.6 on Cyc131 and Cys137, which are close to the catalytic loop and near to Ser175 residues, which is vital for the phosphorylation of SnRK2.6 [133,134,135,136,137]. The Cys137 can also undergo *S*-nitrosylation and could inhibit the activity of SnRK2.6 [9,136]. However, persulfidation promotes SnRK2.6 activity, and it is believed that persulfidation occurs earlier than *S*-nitrosylation [9,137]. Due to Cyc131/137 persulfidation induced changes, Ser175 affinity for ATP-γ-phosphate proton acceptor site (Asp140) increases, which leads to the robust autophosphorylation of Ser175 and triggers efficient interaction of SnRK2.6 with its target. This observation confirms that H_2_S-mediated persulfidation positively impacts the function of SnRK2.6 in ABA-mediated stomatal closure in guard cells [9,135]. Likewise, Shen et al. [138] reported that during drought stress, ABA signaling in guard cells is promoted by H_2_S interaction with ABA. The drought stress mediates the accumulation of ABA, which stimulates persulfidation of DES1 in a redox-dependent manner. At the physiological level, enhanced accumulation of H_2_S in the guard cell leads to the persulfidation of H_2_O_2_ producing enzymes, such as NADPH oxidase, which triggers the generation of H_2_O_2_ in the guard cell that reinforces ABA signaling and the closure of stomata [138]. Another study revealed that abscisic acid insensitive 4 (ABI4) is involved in the facilitation of ABA and H_2_S crosstalk at the transcriptional level (Figure 4). ABI4 is a vital TF in the ABA signaling cascade, and little was known about the PTMs that regulate its activity in response to ABA/H_2_S interaction in plants. The ABA accumulation triggers a massive generation of H_2_S that leads to the persulfidation of ABI4, which allows the binding of ABI4 to the E1 motif of the *MAPKKK18* (*mitogen-activated protein kinase kinase kinase 18*) promoter to activate *DES1* transcription to close stomata under the ABA-dependent signaling cascade [43]. This study provides compelling evidence that the DES1/H_2_S-ABI4 module acts downstream of ABA signaling to regulate stomatal closure [43,139] (Figure 4).

In some of the recently published reports, it was also revealed that H_2_S might be involved in the biosynthesis of ABA in guard cells [140]. The H_2_S promotes the synthesis of cysteine, which is a substrate of ABA3 (molybdenum cofactor sulfurase) enzymes that regulate the activation of AAO3 (abscisic aldehyde oxidase 3) [141]. The higher accumulation of cysteine stimulates the activity of AAO (in vivo) and favors the synthesis of ABA [39] by stimulating the transcript abundance of *NCED3* (9-*cis*-epoxycarotenoid dioxygenase 3). It was revealed that H_2_S could boost ABA synthesis, because in a cysteine-biosynthesis-depleted mutant with the disrupted ABA biosynthesis, the H_2_S was unable to induce stomatal closure [135,136]. All these studies point out the involvement/crosstalk of H_2_S with SnRK2.6, CPK6, MAPKKK18, ABI1, NADPH oxidase, Ca^2+^, and ROS in ABA-mediated signaling for stomatal movements in plants [135,136,137].

### 4.2. Nitric Oxide (NO) and H_2_S: Two Interacting Gaseous Molecules Essential for Plant Functioning

Nitric oxide (NO) is also a lipophilic gaseous hormone that could diffuse into inter- or intra cellular spaces without the need for any carrier or transport channel. NO is also involved in PTMs via tyrosine nitration, metal nitrosylation, and S-nitrosylation, whereas H_2_S mediated-PTM is associated with persulfidation. However, all these reactions led to the modification of structure, localization, and function of target proteins. Several studies have shown that H_2_S interacts with NO and other signaling molecules to modulate plant development and stress responses [7,26,32,34,142]. Earlier reports indicate that the interaction of H_2_S towards NO is complementary or inhibitory [55,143,144,145,146]. The positive or negative interaction of these two gaseous signaling molecules may be dependent upon the dosage of exogenous H_2_S or NO application. For instance, the level of NO was reduced in plant tissues that were treated with H_2_S modulator (NaSH) [126,147]. However, crosstalk of NO-H_2_S showed synergistic interaction during abiotic stresses and inhibition of ethylene-induced fruit ripening, whereas antagonistic interaction of H_2_S-NO-ethylene is also reported [16,148,149,150]. The discrepancy in H_2_S and NO interaction may depend upon the specific location of these gaseous molecules in the cell that deicide their signaling behavior [151]. There is also a possibility that both gaseous molecules may compete for the same targeting protein in the cell. For example, SnRK2.6 is a target of both NO and H_2_S biomolecules, and S-nitrosylation of SnRK2.6 via NO inhibits its activity while persulfidation enhances its activity and mediate stomatal movements [135,137]. Additionally, H_2_S and NO could react among themselves to produce nitrosothiol compounds that are also involved in signaling responses. The crosstalk of ROS with H_2_S–NO cascades also modulates their interactions in positive or negative ways [152]. Taken together, the nature of the interaction between NO and H_2_S may vary for different physiological functions based upon their location and concentration in the cell.

NO and H_2_S belong to the family of reactive nitrogen and sulfur species (RNS and RSS), and their positive combinations regulate various important physiological and molecular processes in plants. For example, the interaction of H_2_S with NO and Ca^2+^ regulate lateral root (LR) formation in tomato plants. The exogenous application of NO triggers the accumulation of H_2_S in tomato roots due to the upregulation of H_2_S biosynthesis enzymes, which induce later root formation [6]. However, when H_2_S inhibitor/scavengers were applied, LRs’ formation was partially arrested. These findings indicate that NO-induced H_2_S synthesis governs the later root formation [6,153].

Stomatal movements are regulated by many endogenous signaling molecules; among them, H_2_S and NO crosstalk are also responsible for stomatal closure. In a recent study, with the employment of pharmacological, spectrophotographic, and fluorescence microscope techniques, the coordinated action of H_2_S and NO in the presence of 2,4-epibrassinolide (EBR) was involved in stomatal regulation [154,155]. The authors demonstrated the application of EBR-induced stomatal closure in a dose and time-dependent manner via modifying the levels of NO, and H_2_S in *Vicia faba*. The application of EBR upregulated the activity of L-/D-cysteine desulfhydrase and enhanced the endogenous levels of H_2_S together with H_2_O_2_ and NO generation in guard cells. The application of the H_2_S inhibitor significantly reduced L-/D-cysteine desulfhydrase activity and H_2_S endogenous production, which in turn abolished the EBR mediated stomatal closure effect [154]. The H_2_S scavengers/inhibitors did not affect the NO and H_2_O_2_ levels in guard cells. However, the application of NO and H_2_O_2_ inhibitors/modulators significantly affected the endogenous production of H_2_S and its biosynthesis enzymes and compromised the EBR-induced stomatal closure [154]. Similarly, Jing et al. [156] found that H_2_S may function downstream of NO in ethylene-induced stomatal closure in *V. faba*. These results indicate that H_2_S and NO participate in EBR-mediated stomatal closure response and H_2_S signifies an essential constituent downstream of H_2_O_2_ and NO in EBR-induced stomatal closure in *V. faba* [154,157]. Previous studies demonstrated that H_2_S inhibits ABA-mediated NO generation in Arabidopsis and *Capsicum annuum* guard cells. Conversely, H_2_S increased NO levels in alfalfa seedlings [55,147], while H_2_S induces NO generation in Arabidopsis guard cells. Conversely, NO scavenger inhibited H_2_S-induced stomatal closure [145]. However, investigation of H_2_S-mediated guard cell signaling in Arabidopsis revealed that the H_2_S induced signaling cascade for stomatal closure is NO-dependent [128], and both H_2_S and NO equally contribute to the production of 8-mercapto-cGMP, which triggers stomatal closure. In the same way, H_2_S and NO collaborate in ethylene induce stomatal closure responses in Arabidopsis plants, and H_2_S generation is mediated by NO, which suggests that H_2_S acts as a downstream signaling agent in ethylene induce stomatal closure [158].

The crosstalk of H_2_S and NO in the alleviation of metal toxicity is also reported, but these studies focused more on stress physiology and lacked underlying molecular mechanisms of crosstalk [159]. The exogenous application of H_2_S donor alleviated Cd stress in alfalfa plants by triggering the synthesis of NO. The interaction mechanism between H_2_S and NO improved the Cd stress tolerance by reducing Cd accumulation and lowering the lipid peroxidation in stressed plants [136]. Another study, where H_2_S and NO scavenger and inhibitor were applied to Cd stressed bermudagrass plants, revealed that depletion of NO makes them more vulnerable to metal toxicity. Furthermore, through pharmacological experiments, it was demonstrated that NO-activated H_2_S was essential for cadmium stress responses in bermudagrass [160]. In *Pisum sativum*, positive interaction of NO and H_2_S was also explored under arsenate stress [109]. The application of H_2_S donor triggered endogenous H_2_S and NO accumulation in *P. sativum*, which led to the strengthening of the antioxidant defense system, reduced arsenate accumulation, and maintained the redox balance of *P. sativum* plant under metal toxicity [109]. Similarly, the crosstalk of NO and H_2_S reduced oxidative stress and increased salinity tolerance in alfalfa, while barley seedlings under H_2_S application regulate ion homeostasis under salinity via maintaining the NO signaling pathway [8,146]. Most of the published studies on the interaction of NO and H_2_S in the context of metal toxicity/salinity proposed that crosstalk of these gaseous molecules ameliorates stress-induced toxicity in exposed plants via (i) improving the antioxidant defense to prevent oxidative stress, (ii) reducing the metal uptake, and (iii) by modulating the expression of associated metal transporter genes [159].

In short, H_2_S and NO are both gaseous biomolecules with common signaling pathways, and it seems that one pathway controls the functions of the other [159]. The persulfidation promoted by H_2_S reacts with thiol groups in the same way as NO does in modification through S-nitrosation [159,161]. However, there is still a need to investigate the interaction of H_2_S and NO in different plant species, tissues, and diverse environmental conditions to unveil the regulatory mechanism of the NO–H_2_S signaling cascade in plants.

### 4.3. H_2_S-Mediated Manipulation of Auxin Signaling in Plants

The development of roots, including lateral and adventitious roots, is incredibly important for normal plant growth and the successful completion of the life cycle. Plant root architecture is mainly based on the LR that is generated from pericycle founder cells [155]. The plant hormone auxin and environmental factors (i.e., water and nutrient availability) are key influencers in lateral root formation [162,163]. Since auxin is a master regulator of root development in plants, there have always been complex crosstalks of auxin with other signaling agents in the root development [162,164,165].

**Figure 5:**
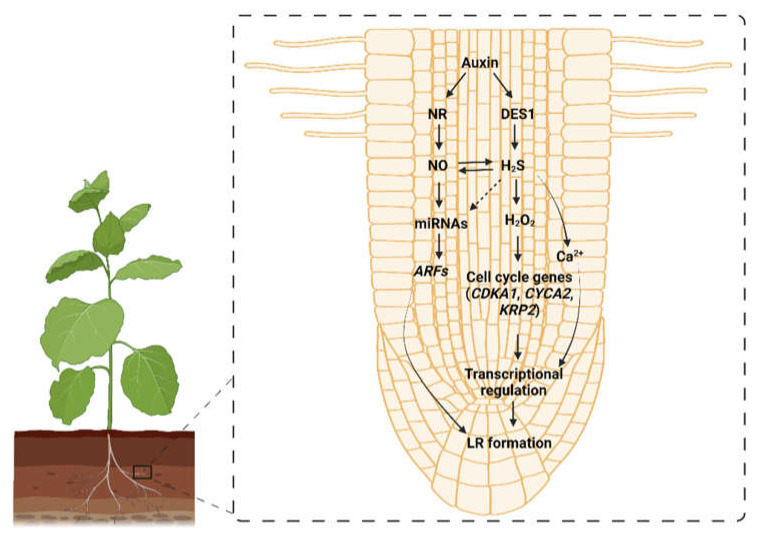
Schematic representation of the signaling pathways involving auxin, DES (cysteine desulfhydrase), NO (Nitric oxide), and hydrogen sulfide (H_2_S) interaction during lateral root formation in plants. The interaction between H_2_S and NO under the influence of auxin participates in the development of the lateral root via modulating the expressions and activities of different effector genes or proteins in a framework of regulatory pathways to permit root growth. miRNA: Micro RNA; ARFs: Auxin Response Factors; *CDKA1*: *Cyclin-Dependent Kinases* gene; *CYCA2*: *cell cycle regulatory* gene; *KRP2*: Kip-Related Protein 2; NR: Nitrate reductase; LR: lateral root.

Several studies have reported that H_2_S and auxin interact with each other to regulate root growth; however, mechanistic insight remains to be elucidated [120,154,166]. The earlier studies demonstrated that the application of exogenous H_2_S on the sweet potato seedling stimulated the numbers and length of adventitious roots by modulating the IAA levels in a dose-dependent manner [154]. It was also noted that pretreatment of H_2_S donor upregulated the transcript abundance of the auxin-dependent Cyclin-Dependent Kinases gene (*CDKA1*) and a cell cycle regulatory gene (*CYCA2*) [153,165]. The activity of both of these genes was inhibited either by auxin blocker or H_2_S inhibitor, which illustrated that H_2_S mediated LR development is dependent upon the IAA signaling via influencing the regulation of *CDKA1* and *CYCA2* [153,165]. Similarly, when higher doses of H_2_S donor (1 mM) were applied, *the RBOH1* (respiration burst oxidase homologous) transcript was significantly upregulated and ROS accumulation triggered the later root formation [115] (Figure 5). The pharmacological studies revealed that H_2_S triggered the expression activity of *RBOH1,* which stimulated an H_2_O_2_-mediated increase in IAA signaling via regulation of *CDKA1*, *CYCA2*, and *Kip-Related Protein 2* (*KRP2*), to activate LR formation [115]. A transcriptomic study revealed that exogenous application of H_2_S impacted the regulation of various auxin pathway-related genes. The accumulation of auxin biosynthesis genes (*TAA1* and *UGT74B1*) was correlated with the increase in auxin levels in roots. The genes involved in auxin polar subcellular distribution, such as *PIN2*, *ABCB1*, *ABCB19*, *PILS3*, and *PILS7*, were differentially expressed, while *PIN1c* appeared as a hub gene on the basis of WGCNA analysis. This study provides sufficient evidence that H_2_S induced root development emanates from regulating the genes involved in transcriptional control and synthesis of auxin [166] (Figure 5).

In some studies, the application of higher dosages of H_2_S showed changes in root development and inhibition of auxin transport due to the alteration in the polar subcellular distribution of the PIN proteins [166]. The polar subcellular movement of auxin in root cells is an actin-dependent process, and H_2_S is involved in the regulation of actin dynamics due to the persulfidation and depolymerization of F actin [167]. Furthermore, during root hair development, the H_2_S fine-tuned polar auxin transport via persulfidation and actin filament growth [167,168]. In the root developmental process, actin-binding proteins work downstream of the H_2_S signal transduction pathway because actin-binding proteins are involved in the depolymerization of F-actin in root cells, which regulate the distribution and transport of auxin [168]. Auxin affects the patterning and organization of the actin cytoskeleton in root cells during cellular growth [169,170]. Conversely, the actin cytoskeleton modulates the directional transport of auxin by altering auxin efflux carriers [171,172]. This finding indicates that overproduction of H_2_S significantly increases the S-sulfhydration level of actin-2 and decreases the distribution of actin cytoskeleton in root cells, thereby reducing auxin’s polar transport, which restricts the LR and the root hair growth [44,167,168].

The exposure of plants to CH_4_ strongly induces H_2_S production and affects the root growth, adventitious root numbers, and root length in cucumber explants [106,173]. At the transcriptional level, it was observed that H_2_S modulated auxin-signaling genes (*Aux22D-like* and *Aux22B-like*) reinforce the CH_4_-induced cucumber adventitious rooting network [111,173,174,175]. Similarly, in tomato plants, LRs formation was also triggered by the CH_4_-mediated H_2_S signaling cascade. It was hypothesized that the possible involvement of auxin transport and auxin signaling in CH_4_-induced LR formation is involved [176]. However, more biochemical and genetic investigations are required to analyze the detailed targets and their functions in root organogenesis under CH_4_-H_2_S-Auxin crosstalks [173,176].

**Figure 6:**
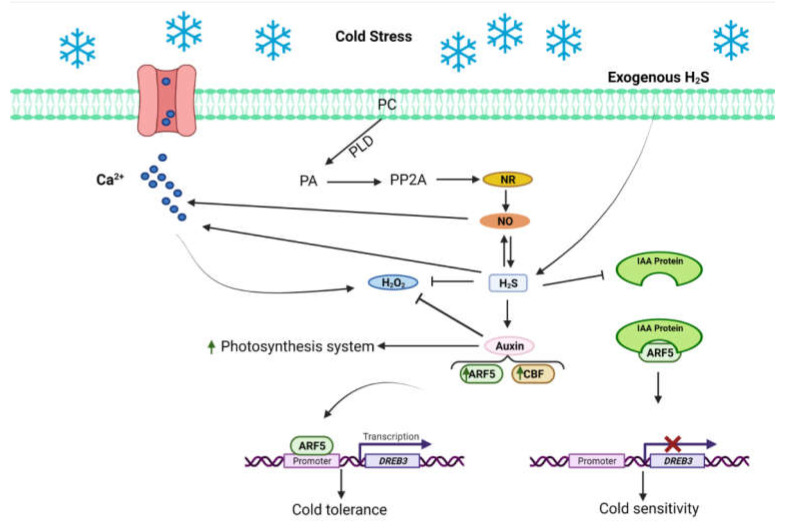
A regulatory model elucidates the role of hydrogen sulfide (H_2_S) in mediating the cold stress response in plants via auxin signaling. In the presence of cold stress, the phospholipase D (PLD) is activated and degrades the phosphatidylcholine (PC) phospholipid of the cell membrane. As a result, phosphatidic acid (PA) is produced, which further regulates protein phosphatase 2A (PP2A), nitrate reductase (NR), nitric oxide (NO), and finally H_2_S. In the absence of H_2_S, auxin distribution, photosynthesis, and carbon assimilation are inhibited in plants under exposure to cold stress. The exogenous application or endogenous H_2_S mediate auxin redistribution in plants and activate the antioxidant defense system along with improved photosynthesis to restore the normal function of the plant at physiological levels. On the other hand, C-repeat binding factors (CBFs) and ARF (auxin-responsive proteins) promote the dehydration-responsive element-binding (DREB) and other related proteins to promote cold tolerance at molecular levels under H_2_S-mediated signaling.

The signaling pathways of H_2_S and auxin interaction under the chilling stress were recently explored in cucumber plants [177,178,179] (Figure 6). The study demonstrated that chilling stress in cucumber arrested photosynthesis and induced oxidative stress; however, deleterious effects were alleviated due to exogenous application of H_2_S donor or IAA application [179]. The expression of *YUCCA2* (auxin biosynthesis gene) and auxin contents were very high in chilling-exposed cucumber seedlings. This result may be due to the inhibition of polar transport of IAA in long-term chilling stress, which increases auxin concentration in leaves and inhibits plant growth. The complex interaction of H_2_S and IAA under chilling stress improved the activities and gene expression of key enzymes of the Calvin–Benson cycle (Ribulose-1,5-bisphosphatecarboxylase, fructose bisphosphatase, sedoheptulose-1,7-bisphosphatase, fructose-1,6-bisphosphate aldolase, and transketolase) and strengthened the photosynthetic carbon assimilation capacity [179] (Figure 6). The results also indicated that auxin is a downstream signal for the protective effects induced by H_2_S under chilling-induced tolerance in cucumber plants [179]. Furthermore, the overexpression of *auxin response factor 5* (*ARF5*) in cucumber unveiled the molecular mechanism of cold tolerance. In transgenic plants overexpressing *ARF5* under cold stress, ARF5 directly activates the expression of *dehydration-responsive element-binding protein 3* (*DREB3*) for the reinforcement of auxin signaling to improve cold stress tolerance in cucumber in response to H_2_S application [180] (Figure 6). Previously, it was observed that *auxin response factors* (*ARFs*) and miR390 formed an auxin-responsive regulatory network (miR390-TAS3-ARF2/ARF3/ARF4) that strengthens auxin signaling in plants [181].

### 4.4. Interaction between H_2_S and Gibberellic Acid

Gibberellic acid (GA) is a phytohormone that substantially influences the seed germination and growth of seedlings. Imbibition of barley grains in 0.25 mM NaHS solution caused an upsurge in antioxidant enzymes such as CAT, POD, APX, and SOD in the aleurone layer [182]. In tomato plants, boron stress reduced dry weight, photosynthetic rate, water content, chlorophyll content, and increased H_2_O_2_, MDA, and endogenous H_2_S. GA foliar spray reduced the harmful effects of boron by raising endogenous H_2_S, Ca^2+^, and K^+^, as well as lowering the levels of H_2_O_2_, MDA, and boron, as well as membrane leakage. Surprisingly, NaHS further increased GA-induced boron tolerance, whereas H_2_S scavengers prevented it (HT). These findings indicate that H_2_S plays a signaling role downstream of GA in the development of boron stress tolerance in tomato plants. During cadmium stress, the NaHS treatment stimulated the activities of amylase and antioxidant enzymes in cucumber hypocotyls and radicles, which might be connected to H_2_S-induced Cd stress tolerance.

Moreover, GA can cause programmed cell death (PCD); however, NaHS application can prevent PCD by lowering L-cysteine desulfhydrase (LCD) activity and accumulating endogenous H_2_S in wheat aleurone layers [49]. GA-induced PCD is reduced in the aleurone layer in the NaHS-treated seeds by diminishing the endogenous GSH levels. H_2_S concentration regulates the GSH levels, which upsurges expression of the *HEME OXYGENASE-1* (*HO-1*) gene, resulting in the alleviation of apoptosis in the aleurone layer and an overall decrease in PCD. Hence, in the aleurone layer, there are regulatory interactions between GA, H_2_S, GSH, and HO-1. Intriguingly, NaHS pretreatment slowed Arabidopsis seed germination, but Arabidopsis *des1* mutant seedlings were more susceptible to ABA than the wild-type. These findings suggest that H_2_S interacts with GA in plants to control seed germination under normal and stressful circumstances.

### 4.5. Interaction between H_2_S and Melatonin

Melatonin (N-acetyl-5-methoxytryptamine) is a multifaceted phytohormone involved in germination, ripening, flowering, photosynthesis, and defense mechanisms [183]. In plants, melatonin alters the permeability of the cell layer governed by ion transporters, which control stomatal opening and closure. Studies have shown that melatonin can increase the photosynthetic capacity of plants, which leads to greater levels of nitrogen and chlorophyll. In tomato and wheat, increased transcription of stress-responsive genes was induced by melatonin, resulting in better tolerance to high temperature [184,185]. Furthermore, melatonin cross-talks with various plant hormones and signaling molecules. It was also discovered that H_2_S and melatonin conjointly helped alleviate salt stress-induced growth reduction in tomatoes, and exogenous melatonin treatment assisted in regulating early H_2_S signaling [186]. In wheat, the heat stress-induced oxidative damage was mitigated by exogenous melatonin and further increased the H_2_S production, suggesting that melatonin-mediated H_2_S was involved in alleviating the oxidative stress. However, the melatonin function was attenuated when H_2_S was inhibited by its inhibitor, indicating that the cross-talk between H_2_S and melatonin, and possibly melatonin, regulates heat stress signaling by acting upstream of H_2_S [187].

## 5. H_2_S-Plant Hormone Cross-Talk under Pathogen Attack

In plants, the dual roles of H_2_S in interactions with phytohormones determine the biological roles of H_2_S in plant growth, development, and responses to biotic stresses. In response to biotic stresses, the crosstalk between H_2_S and phytohormones, as well as several other signaling molecules, has been studied less; however, some critical molecular insights have been found in the recent past. In the following paragraph we discuss the H_2_S–phytohormone interplay under biotic stress.

### 5.1. Interaction between H_2_S and Salicylic Acid

Salicylic acid (SA) is a phytohormone that triggers a defense response in plants against biotrophic and hemibiotrophic phytopathogens. SA activates a large number of defense-related genes, especially those that encode pathogenesis-related (PR) proteins [188,189]. Susceptibility to virulent and avirulent pathogens develops as a result of mutations that impede SA production. In *Nicotiana tabacum* cv. Xanthi-nc, acetyl SA (aspirin) confers resistance to tobacco mosaic virus [190]. Previously, it was found that the expression of multiple WRKY transcription factors (TFs) is modulated by pathogen attack or SA treatment [191]. A subsequent study has shown that the mutation in *WRKY18*, *WRKY40*, and *WRKY60* resulted in the up-regulation of *LCD*, *DES*, *DCD1*, and higher production of H_2_S in Arabidopsis [192]. In Arabidopsis, the expression level of a *PR* gene-regulating transcription factor *WRKY54* was elevated in *des1* mutants and decreased in *oas-a1* mutants [193]. Furthermore, *des1* mutants had lower levels of L-glutathione oxidation than *oas-a1* mutants, and lesser intracellular redox potential was caused by higher L-Cys levels in *des1* mutants, which may help boost plant resistance to pathogen invasion [193]. Later, Alvarez et al. [194] demonstrated that Arabidopsis *des1* mutants have increased amounts of SA and developed more resilience against *Pseudomonas syringae* pv. *tomato* (*Pst*) DC3000 *avrRpm1*, while *oas-a1* mutants were more vulnerable to this pathogen [194]. The *des1* mutants exhibited all the constitutive systemic acquired resistance characteristics, including high resistance against biotrophic and necrotrophic pathogens, accumulation of salicylic acid, and induction of *WRKY54* and *PR1* [194]. In contrast to the *oas-a1* mutants, Arabidopsis *cad2-1* mutants showed lower levels of L-glutathione but a non-significant change in the L-Cys levels. In *cad2-1* mutants, repression of *WRKY54* was also not observed, which suggests that lower expression of *PR* genes in *oas-a1* mutants might be due to reduced L-Cys level [192]. In order to determine if L-Cys is involved in plant immunity, researchers exposed *oas-a1* mutants to the bacterial pathogen *Pst* DC3000, which releases effectors that suppress PAMP-triggered immunity (PTI). The Arabidopsis *oas-a1* mutant plants were shown to be more susceptible to infection by this pathogen [195]. Thus, the results from the previously mentioned studies suggest that higher L-Cys decreases cytoplasmic redox potential, which may play a key role in pathogen defense in Arabidopsis and other plant species. Still, more research is needed in Arabidopsis and other plant species.

**Figure 7:**
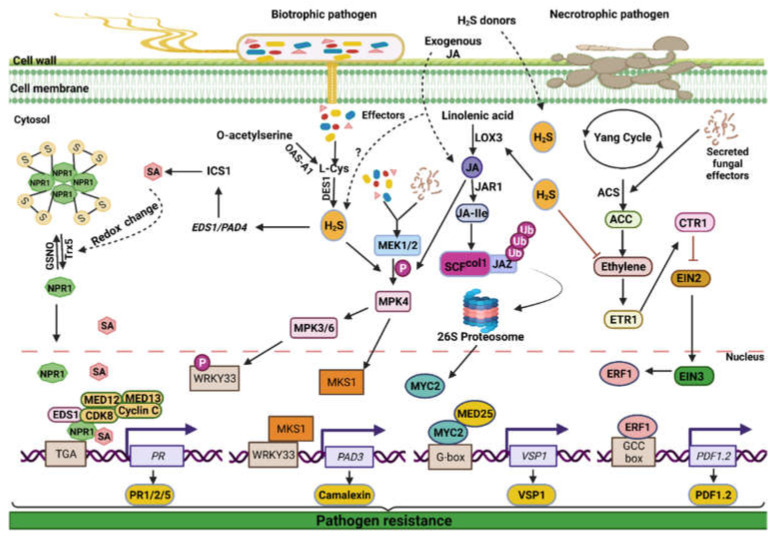
A schematic model of the cross-talks between H_2_S and salicylic acid (SA), jasmonic acid (JA), and ethylene (ET) in plant defense against pathogens. The biotrophic pathogen attacks plants and secretes effectors into plant cells. The conversion of O-Acetylserine into L-Cysteine (L-Cys) is catalyzed by Anthranilate synthase (OAS-A1). Similarly, effectors also induce the biosynthesis of L- Cys. The plant cytosol contains the enzyme L-cysteine desulfhydrase (DES1), which is responsible for L-Cys decomposition and endogenous H_2_S production. The higher concentration of H_2_S triggers the upregulation of SA biosynthesis-related genes (*PAD4*/*EDS1*). The enzyme ICS1 catalyzes the conversion of chorismite into isochorismate, which is then exported to the cytosol by EDS5. The L-glutamate is converted into isochorismate-9-glutamate in the cytosol by PBS3. Subsequently, SA is produced from isochorismate-9-glutamate through spontaneous decay. By acting as an isochorismate A pyruvoyl-glutamate lyase (IPGL), EPS1 also degrades N-pyruvoyl-L-glutamate to create SA. The *NPR1* gene expression is aided by SA due to the interaction of WRKY transcription factors with NPR1, which promotes the recruitment of CDK8 to the *NPR1* promoter’s W-box. Pathogen-induced defense signals enhance the accumulation of salicylic acid (SA) in plants by enhancing the expression of *Isochorismate Synthase* (*ICS*) genes. In addition, SA promotes redox reactions that lead to the reduction of NPR1 oligomers to monomers. The monomeric NPR1 molecules move from the cytosol to the nucleus, where they form a protein complex with transcription factor (TGA), EDS1, SA, and CDK8, resulting in the transcription of *PR* genes. A higher concentration of H_2_S upregulates the JA biosynthetic gene *LOX3*. Moreover, the exogenous application of JA also increases the endogenous H_2_S and JA. The secreted effectors by biotrophic and necrotrophic pathogens trigger the pattern recognition receptors (PRR), which further activate the plant mitogen-activated protein kinase (MEK1/2) cascades. H_2_S and JA participate in phosphorylation of MEK1/2, subsequently triggering MPK4. The MPK4 activates the MPK3/MPK6 and MKS1 (the substrate of MPK4). WRKY33 is involved in the biosynthesis of camalexin (a phytoalexin). MPK3/MPK6 phosphorylate the WRKY33 and increase its transactivation activity. The WRKY33 forms a complex with MKS1 for the transcription of *PAD3*, which activates the biosynthesis of camalexin. In the elicited cells, JA-Ile COI1, an F-box protein in the SCF ubiquitin E3 ligase complex, recognizes JA-Ile and facilitates the binding between COI1 and the JAZ family of repressor proteins, resulting in JAZs being ubiquitinated. The 26S proteasome then degrades the ubiquitinated JAZs. JAZ degradation promotes downstream JA responses by releasing the target transcription factor (MYC2) from inhibition. The Mediator25 binds to the MYC2 to enhance the transcriptional activity of wound-responsive gene *VSP1*. H_2_S molecules work as a repressor for ethylene signaling. In response to the effectors of the necrotrophic pathogen, the ethylene biosynthesis genes 1-aminocyclopropane-1-carboxylic acid synthase (ACS), 1-aminocyclopropane-l-carboxylic acid (ACC) are activated, resulting in the formation of ethylene. Under normal growth conditions with low ethylene levels, the Ethylene receptor 1 (ERT1) remains in the active state and associates with CTR1, which, in turn, inhibits the downstream signaling pathway. The ethylene binding inactivates its receptors and in turn deactivates the Raf-like kinase CTR1. Consequentially, EIN2 can function and signal positively downstream to the ethylene insensitive 3 (EIN3) of transcription factors situated in the nucleus. EIN3 drives the expression of ethylene response factor (*ERF1*). Subsequently, the ERF1 binds to the GCC box and invokes the *PDF1.2* defense gene.

Among SA-biosynthesis genes in Arabidopsis, the phytoalexin deficient (*PAD*) genes (*PAD1*, *PAD2*, *PAD3*, and *PAD4*) encode regulatory proteins that function against the eukaryotic biotroph *Peronospora parasitica* and promote resistance to downy mildew [196]. Increased sensitivity to the bacterial pathogen *Pst* DC3000 has been observed in the *pad1*, *pad2*, and *pad4* mutants [196]. Enhanced disease susceptibility1 (*EDS1*) gene codes for a lipases-like protein that acts in resistance (*R*) gene-dependent effector-triggered immunity and contributes to basal defense in plants. EDS1 is also required for pathogen-induced *PAD4* mRNA accumulation [197]. The *PAD4* and *EDS1* genes involved in SA biosynthesis were found to be constitutively activated in Arabidopsis plants with high H_2_S concentrations but found to be reduced in plants with low H_2_S levels (Figure 7) [58]. NPR1 plays an essential function in SA signaling because it binds SA and initiates a SAR response [198,199,200]. Other similar molecules such as methyl salicylate (MeSA) or gentisic acid promote *PR1* expression in addition to SA [201]. The deposition of SA is required for triggering the expression of SA-mediated genes, such as *PR*s [189]. Plants with greater H_2_S levels showed increased expression of SA-mediated *PR* genes, which improved pathogen resistance, and vice versa (Figure 7).

### 5.2. Interaction between H_2_S and Jasmonic Acid

Jasmonic acid (JA) is a lipid-derived signaling molecule that plays a significant role in many biological processes in plant cells. Herbivorous insects chewing on the leaves or necrotrophic diseases trigger the JA response pathway. Plants have evolved to remember these attacks and employ this pre-conditioned situation effectively and to their benefit in a mechanism termed induced systemic resistance (ISR). Interestingly, the biological pathways of JA and SA have been reported to function antagonistically [202]. JA and SA enhance plant defense against nematodes such as *M. incognita* [203]. This pathogen causes plants to trigger SA pathways and prevent JA in leaves to permit successful invasion of the pathogen. Furthermore, JA showed a higher concentration in roots following the nematodic infection that is subsequently transferred to leaves, helping plants to defend themselves against pathogens [204]. In another study, when Arabidopsis was deprived of the sulfur element, it led to activation of the JA and SA metabolism; but the plant showed susceptibility to necrotrophic *Botrytis cinerea* [205]. This discovery suggests that the presence of sulfur-containing compound H_2_S is essential for plant defense mechanisms through its interaction with SA and JA.

H_2_S interacts with JA to promote pathogen resistance in plants (Figure 7). The redox state of ascorbate is shown to be regulated in the leaves of *A. thaliana* by the interaction between H_2_S and mitogen-activated protein kinase (MEK1/2) (Figure 7) [206]. In Arabidopsis, the exogenous application of JA resulted in a significant increase in endogenous H_2_S generation, MEK1/2 phosphorylation, and a lower ascorbate to dehydroascorbate ratio (AsA/DHA) [195]. The increase in the phosphorylation level of MEK1/2, endogenous H_2_S generation, and the AsA/DHA ratio in wild-type hosts was shown to be caused by hypotaurine (HT), an H_2_S scavenger, resulting in a decrease in JA. The application of sodium hydrosulfide, which acts as an H_2_S donor in mutant *A. thaliana* plants, was observed to enhance these indicators. When these mutant plants were given an application of NaHS after being treated with HT and JA, the effects of hypotaurine on those JA-induced indicators were not reversed.

### 5.3. Interaction between H_2_S and Ethylene

Phytohormones play a critical role in the defense mechanism in plants against various pathogens. SA often controls biotrophic and hemibiotrophic pathogen defense responses, but ethylene and JA promote defense responses to necrotrophic pathogens. However, sometimes hormone signal transduction pathways that conferred resistance and vulnerability were found to be diametrically opposed. Plant resistance was shown to be associated with an increase in SA signaling, whereas susceptibility was found to be associated with an increase in the ethylene pathway and a decrease in SA and cytokinin signaling. According to Foucher et al. [207], two *Phaseolus vulgaris* L. genotypes (resistant and susceptible) were screened against common bacterial blight caused by *Xanthomonas phaseoli* pv. *phaseoli*. The transcriptomic study revealed that resistance was associated with an increase in the SA pathway and a decrease in photosynthetic activity as well as sugar metabolism. Susceptibility was associated with an increase in the ethylene pathway and genes that modify cell walls, as well as a decrease in the downregulation of resistance genes [207].

Pathogenic bacteria cannot form merism when exposed to exogenous NaHS, which helps plants recover from infection [208]. Fumigation with H_2_S has been shown to suppress spore germination, mycelial growth, and pathogenicity of *Monilinia fructicola* in peach fruit, as well as *Aspergillus niger* and *Penicillium expansum* in pear [209]. These findings show that H_2_S can promote a plant’s resistance to pathogen infection, and that immunological signals and exogenous sulfide can both trigger the production of endogenous H_2_S. Exogenous H_2_S reversed the impacts of ETH by reducing the activity of enzymes involved in cell wall modification (cellulase and polygalacturonase) via transcription suppression rather than direct post-translational modification (sulfhydration) by H_2_S [210]. H_2_S also controlled the expression of *SlIAA3*, *SlIAA4*, *ILR-L3*, and *ILR-L4* (all of which are involved in auxin signaling), which suppressed petiole abscission by controlling the amount of free auxin in tomato abscission zone cells. In rose and lily plants, similar findings were observed in floral organ abscission and anther dehiscence [210]. These findings suggest that H_2_S interacts with ethylene and auxin during plant organ abscission.

Exogenous ethylene donor (ethephon) stimulated the activities of LCD and DCD in Arabidopsis and *Vicia faba* plants, resulting in H_2_S production in guard cells and stomatal closure, whereas H_2_S-synthesis inhibitors (PAG) reversed ethylene-induced stomatal closure, indicating H_2_S-mediated ethylene-induced stomatal closure [211]. Furthermore, early leaf senescence was seen in Arabidopsis *des1* mutants (due to reduced endogenous H_2_S content), whereas NaHS treatment reversed the senescence and extended the vase life of cut flowers by elevating endogenous H_2_S levels. In addition, by reducing ethylene synthesis, H_2_S-delayed senescence was seen in green leafy crops [212]. These findings demonstrate that ethylene promotes stomatal closure and organ senescence in plants by independently increasing and suppressing endogenous H_2_S generation.

## 6. Conclusions and Future Prospects

For a long time, H_2_S was considered an undesirable by-product of sulfur metabolism, which could adversely affect plant cells. However, this perception was altered after it was discovered that H_2_S could have signaling properties. H_2_S is involved in many plant processes and can interact with other phytohormones to mitigate stress in plants. However, most research is focused on the H_2_S interaction with phytohormones under abiotic stress. In contrast, there is very limited research progress on the interaction of H_2_S with SA, JA, and especially, ethylene in plants under biotic stresses. The exogenous ethylene donor (ethephon) stimulated the activities of LCD and DCD in Arabidopsis and *V. faba* plants, resulting in H_2_S production in guard cells and stomatal closure, whereas H_2_S-synthesis inhibitors (PAG) reversed ethylene-induced stomatal closure, indicating H_2_S mediates ethylene-induced stomatal closure [211]. Since ethylene promotes stomatal closure, it might prevent the invasion of pathogens. Therefore, it is likely the crosstalk between H_2_S and ethylene plays a pivotal role in the regulation of stomatal closure during plant defense against pathogen invasion, which warrants further investigation.

In plants, the H_2_S-mediated persulfation can significantly impact protein function, altering protein conformation and regulating protein activity under stress response. According to Chen et al. [135], H_2_S positively regulates abscisic acid signaling by sulfidating SnRK2.6 in guard cells. H_2_S has also been reported to persulfidate MAPK in Arabidopsis to alleviate cold stress [213]. Numerous studies have been conducted to understand H_2_S-mediated persulfation of proteins in plants under abiotic stress; however, H_2_S-mediated persulfation is not studied sufficiently in plant–pathogen interaction. H_2_S can also be involved in protein functions through trans-persulfidation and regulating cellular redox state in other unexplored H_2_S-related molecules in the plant metabolism such as glutathione persulfide (GSSH) and cysteine persulfide (CysSSH).

In future studies, more fundamental research is required to investigate the fate and regulation of endogenous H_2_S production, and its subsequent interaction with and regulation of different plant processes under laboratory as well as in field conditions. However, the exogenous application of H_2_S on plants in controlled conditions has generated plenty of experimental results that have explained at least some of the underlying mechanisms of actions driven by H_2_S molecules in plants. In the animal field, several exogenous sources of H_2_S have been utilized that can slowly release H_2_S in media (mimicking the natural generation of H_2_S). However, for plants, NaHS and inorganic sodium polysulfides (Na_2_S_n_) such as Na_2_S_2_, Na_2_S_3_, and Na_2_S_4_ are currently used in various research reports to study the H_2_S impacts in plants. The NaHS and related H_2_S generation compounds are usually short-lived donors and do not mimic the slow release of H_2_S in in-vivo conditions. Recently, dialkyldithiophosphate demonstrated the potential to release H_2_S slowly and enhance the maize plant biomass upon application [214]. In addition, more precise and advanced methods of H_2_S application to the plants under various growth stages and environmental stresses, and H_2_S suitable dosages for different crop species are also required.

## Data Availability

Not applicable.
